# Carbonate chemistry seasonality in a tropical mangrove lagoon in La Parguera, Puerto Rico

**DOI:** 10.1371/journal.pone.0250069

**Published:** 2021-05-05

**Authors:** Erick M. García-Troche, Julio M. Morell, Melissa Meléndez, Joseph E. Salisbury

**Affiliations:** 1 Department of Marine Sciences, University of Puerto Rico at Mayagüez, Mayagüez, Puerto Rico; 2 Caribbean Coastal Ocean Observing System, NOAA-IOOS, Lajas, Puerto Rico; 3 School of Ocean and Earth Science and Technology (SOEST), University of Hawai’i at Manoa, Honolulu, Hawai’i, United States of America; 4 Ocean Process Analysis Laboratory, Institute for the Study of Earth, Oceans, and Space, University of New Hampshire, Durham, New Hampshire, United States of America; Auckland University of Technology, NEW ZEALAND

## Abstract

We investigated the seasonal carbonate chemistry variability within a semi-enclosed tropical mangrove lagoon in southwestern Puerto Rico. Biweekly measurements of seawater temperature, salinity, total alkalinity (TA), and dissolved inorganic carbon (DIC) were conducted from 2014 to 2018. We describe the possible mechanisms driving the observed variability by correlating the DIC/TA ratio with pH and Ω_arg_, suggesting that the mean pH (7.87 ± 0.09) and aragonite saturation state (Ω_arg_, 2.96 ± 0.47) of the mangrove lagoon negatively affected calcification. The measured *p*CO_2_ and DIC/TA ratios indicate that heterotrophic activity was the primary driver for persistent acidification, which reached its maximum expression during the wet season. We conclude that mangrove lagoons with limited seawater exchange and high carbon input will not mitigate ocean acidification.

## Introduction

There is an increasing research interest to identify ecosystems where vulnerable species could persist under foreseen future scenarios, including high atmospheric carbon dioxide (CO_2_) concentrations and seawater temperatures [1]. Surface oceans have already warmed [2, 3] and acidified worldwide at an unprecedented pace [4], and scientists expect such trends to continue based on the Intergovernmental Panel on Climate Change (IPCC) CO_2_ emission scenarios [5].

Studies suggest that different coastal ecosystems, such as seagrass beds [6] and mangroves [7, 8], can mitigate ocean acidification (OA) driven by oceanic uptake of anthropogenic CO_2_. A buffering effect may occur when “*alkalinity increases the CO*_*2*_ *uptake capacity of seawater by neutralizing H*^*+*^ *ions and buffering the pH change associated with CO*_*2*_ *inputs*” [9]. Seagrass ecosystems buffer OA at shallow depths and reduced water mass turnover [10, 11] by raising the pH and calcium carbonate (CaCO_3_) saturation while they are in a state of net autotrophy [10]. The buffering effect may be limited to specific periods throughout the year [12] and could have the opposite effect by exacerbating OA in the future [13].

A Caribbean mangrove system (St. John, United States Virgin Islands) was recently identified as a potential refuge from OA, upper-ocean warming, and solar radiation for non-reef scleractinian coral species [8]. The authors suggested that CaCO_3_ sediment dissolution could have increased downstream total alkalinity (TA) to prevent OA from exacerbating [8]. Moreover, a study in an Australian mangrove creek suggested that mangrove sites could generate alkalinity via sulfate reduction and exported to nearby coastal areas, creating a zone buffered against OA [7]. Other studies suggest that mangrove ecosystems can pre-condition corals to low pH’s [14] and serve as reservoirs for already tolerant coral species [15, 16].

Mangrove communities are known for their high productivity [17, 18] and are more productive than most terrestrial and marine communities [19]. However, global budgets still fail to fully quantify the amount of carbon fixated by mangrove vegetation, given that datasets and estimates on wood production and belowground allocation are limited [20]. Estimates note that inorganic carbon produced by mineralization activity could account for the missing carbon in global budgets, given that mineralization rates are severely underestimated [20]. Sulfate reduction and aerobic respiration are commonly the dominant metabolic pathways within tropical mangrove sediments [7, 21, 22]. The former yields alkalinity, assuming that the resulting sulfide is not re-oxidized to sulfate [23]. Coupled denitrification/nitrification and ammonification are restricted pathways in tropical mangrove environments due to scarce oxygen availability [24–26].

Studies published to date provide a good understanding of the carbonate chemistry diel variability at these mangrove sites [7, 8, 14–16]. However, the buffering role of mangroves at seasonal time scales remains unclear. This study’s main objective is to determine if the tropical mangrove lagoon located on the southwest coast of Puerto Rico can buffer OA. We also aim to assess TA’s seasonal variability, dissolved inorganic carbon (DIC), and physical dynamics (temperature, salinity, precipitation, tides, and winds). We hypothesize that mangrove lagoons with high mangrove carbon mineralization activity and limited flushing provide conditions that promote OA and reduce the aragonite saturation state (Ω_arg_).

## Methods

### Site description

**Fig 1:**
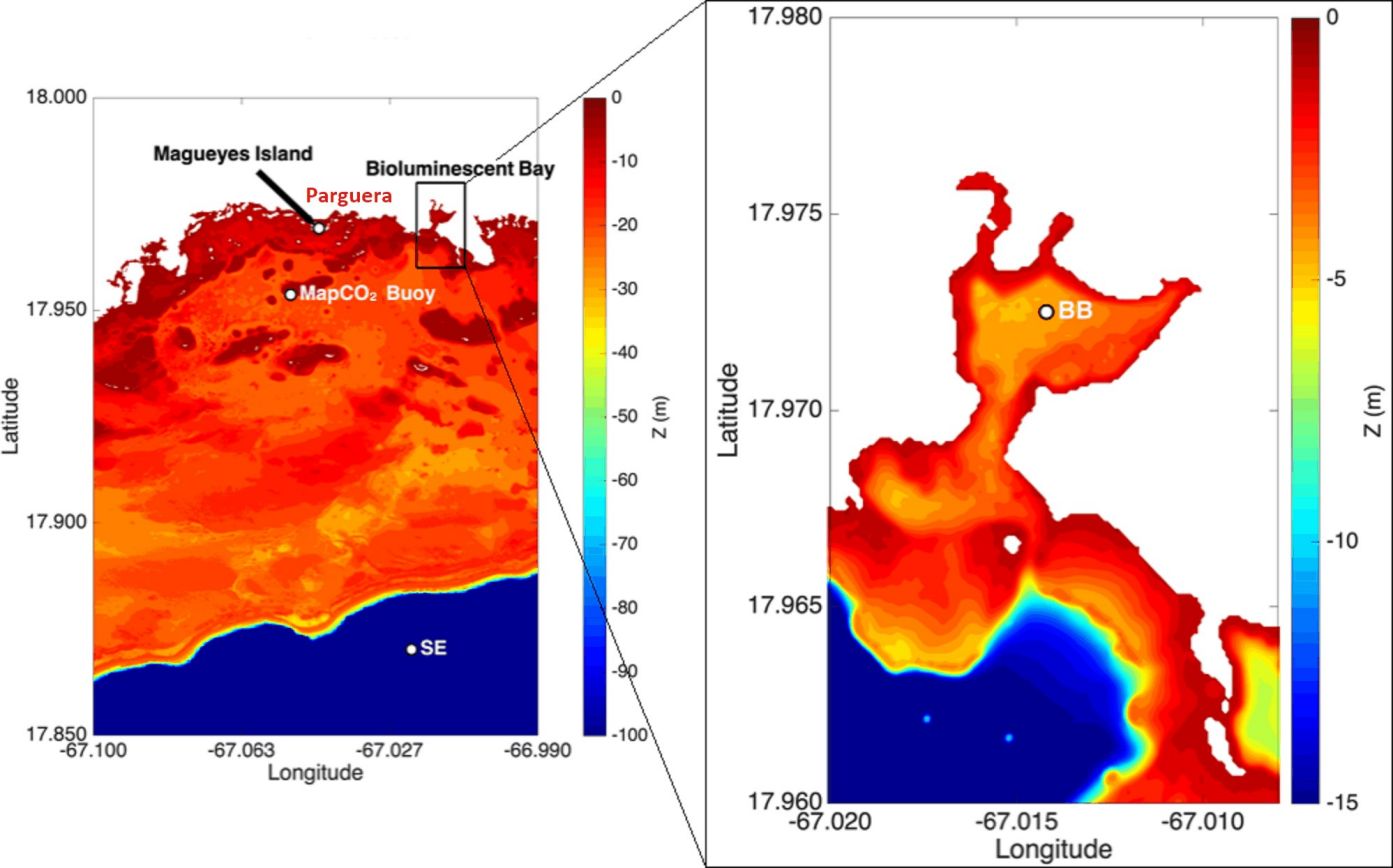
Bathymetric maps of La Parguera Marine Reserve (Lajas, PR) and study sites. La Parguera Marine Reserve is on the left panel. The white dots represent the shelf-edge station (SE), the moored autonomous carbon dioxide partial pressure (Ma*p*CO_2_) buoy, and the Marine Station (Magueyes Island). The Bioluminescent Bay is to the right, and the white dot represents the sampled station (BB). Note the corresponding depth color legend to the right of each map.

The study area is within La Parguera Marine Reserve, an estuary in which evaporation exceeds freshwater influx [27], located on the southwestern coast of Puerto Rico (Fig 1). The Reserve’s cays, submerged reefs, and mangroves ecosystems surround and protect La Parguera town (17.974 ^o^N, 67.046 ^o^W) from intense wave energy. The wet season extends from August to November, while semiarid conditions prevail during the rest of the year (dry season). The Caribbean Surface Water, which constitutes the oceanic end-member for La Parguera, is a mixture of Tropical North Atlantic surface waters and continental river waters [28]. River plumes from the Amazon and Orinoco Rivers impinge in the region [29, 30], with maximum influence from June through October [33, 34]. The area is under the influence of easterly trade winds and a diurnal tide (S1 Fig) of small amplitude (ca. 0.3 meters).

The SE station (17.870 ^o^N, 67.021 ^o^W) is approximately 11 km off the coast of La Parguera and 1 km off the insular shelf-edge (Fig 1). This station represents the oceanic reference end-member.

**Fig 2:**
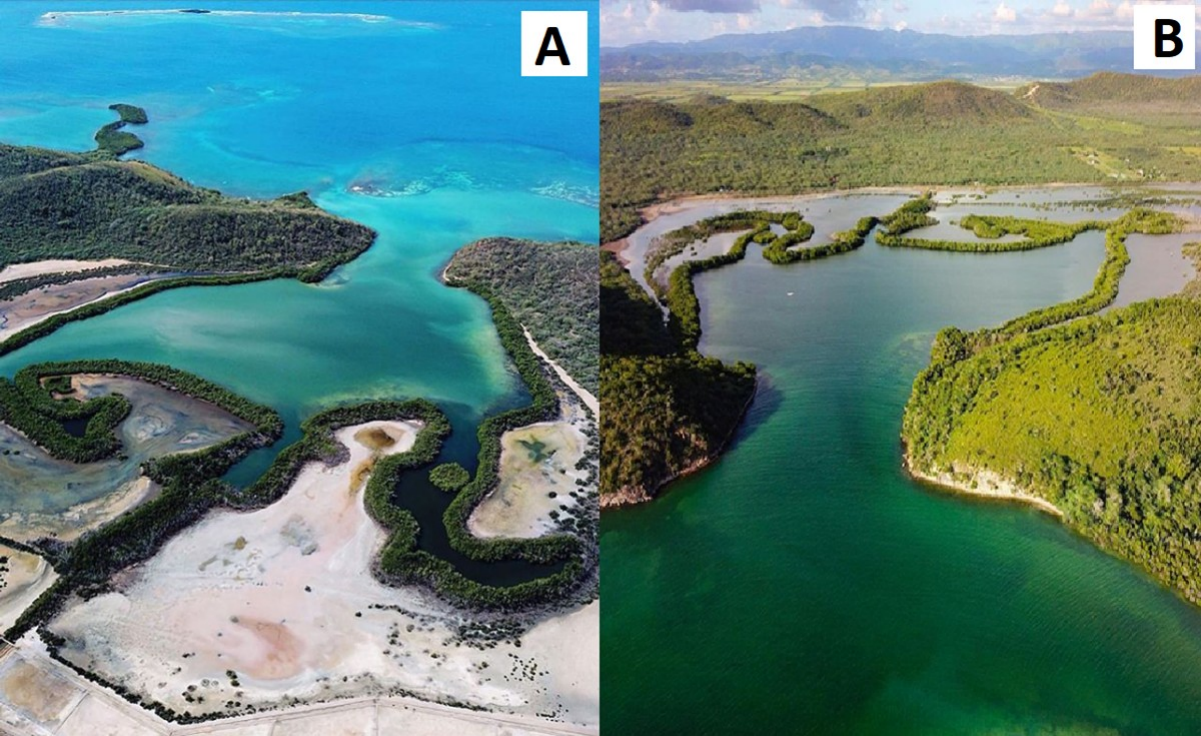
Drone pictures of The Bioluminescent Bay. The picture to the left (A, by Jonathan Vegilla) was shot from the north during the dry season. The picture on the right (B, by Omar López) was taken from the south during the wet season. Note the salt flats to the north of the bay, mostly dry in (A) and flooded in (B).

The BB station (17.972 ^o^N, 67.014 ^o^W) is within The Bioluminescent Bay (Fig 2), 3.2 km east of La Parguera town (Fig 1). The Bioluminescent Bay has a 0.19 km^2^ surface area, an average depth of 3.5 m [31], and a maximum of 4.5 m [32]. A 25-meter-wide red mangrove (*Rhizophora mangle* L.) fringe surrounds the bay and is a significant organic carbon source. The total estimated primary productivity for *R*. *mangle* in La Parguera was 3.85 g dry wt m^-2^ day^-1^, while the litterfall fraction was in the 0.15 to 0.91 g dry wt m^-2^ day^-1^ range with a marked seasonal variability [33]. The BB water column’s net community production has been reported to range from -5.99 to -2.49 g C m^-2^ day^-1^ [34, 35]. The bathymetry has an irregular shape with a narrow and shallow outlet to nearby shelf waters and a sill (1 m depth) at the entrance, limiting exchange with outer waters and shelters from wave energy [32, 36]. Most of the bottom comprises muddy sediments (ca. 7.5% grams of organic carbon content per gram of sediment, down to a 20 cm depth [37]). While coral reef systems are not known to exist within the bay, small coral communities live 200 meters beyond its entrance. Runoff from salt flats to the north of the bay flows into the lagoon via three channels during infrequent heavy rain periods. The lagoon is partially sheltered from the prevailing trade winds by mangroves and hills on its outlet’s east and west sides.

### Fieldwork

Surveys were conducted every two weeks at SE (June 2014 to May 2018) and at BB (June 2014 to May 2015, October 2016 to May 2018) stations at approximately 8:00 and 9:00 AM local time, respectively. At times, we altered sampling frequency due to unfavorable weather conditions or instrument failure. Sampling depth at the SE station was 4 m, while at BB, it was 3 m. No permits were required to conduct this fieldwork.

Our data set includes DIC and TA measurements from discrete seawater samples, drawn from a 2.2 L Van Dorn sampler into 250 (or 125) mL borosilicate glass bottles and secured using greased stoppers, following the guide for best practices for CO_2_ measurements [38]. Each sample was fixed immediately with 0.02% of the total bottle volume using a saturated solution of mercury chloride (HgCl_2_) or immediately after returning to the laboratory (same day) to prevent biological alteration and stored at room temperature for later analysis. Each bottle was tightly sealed with about 1% of headspace to prevent atmospheric gas exchange. Analyses were performed within six months after sampling. Profile measurements of temperature and salinity were taken using an SBE25® conductivity, temperature, and depth recorder (CTD).

### Meteorological and tidal observations

**Fig 3:**
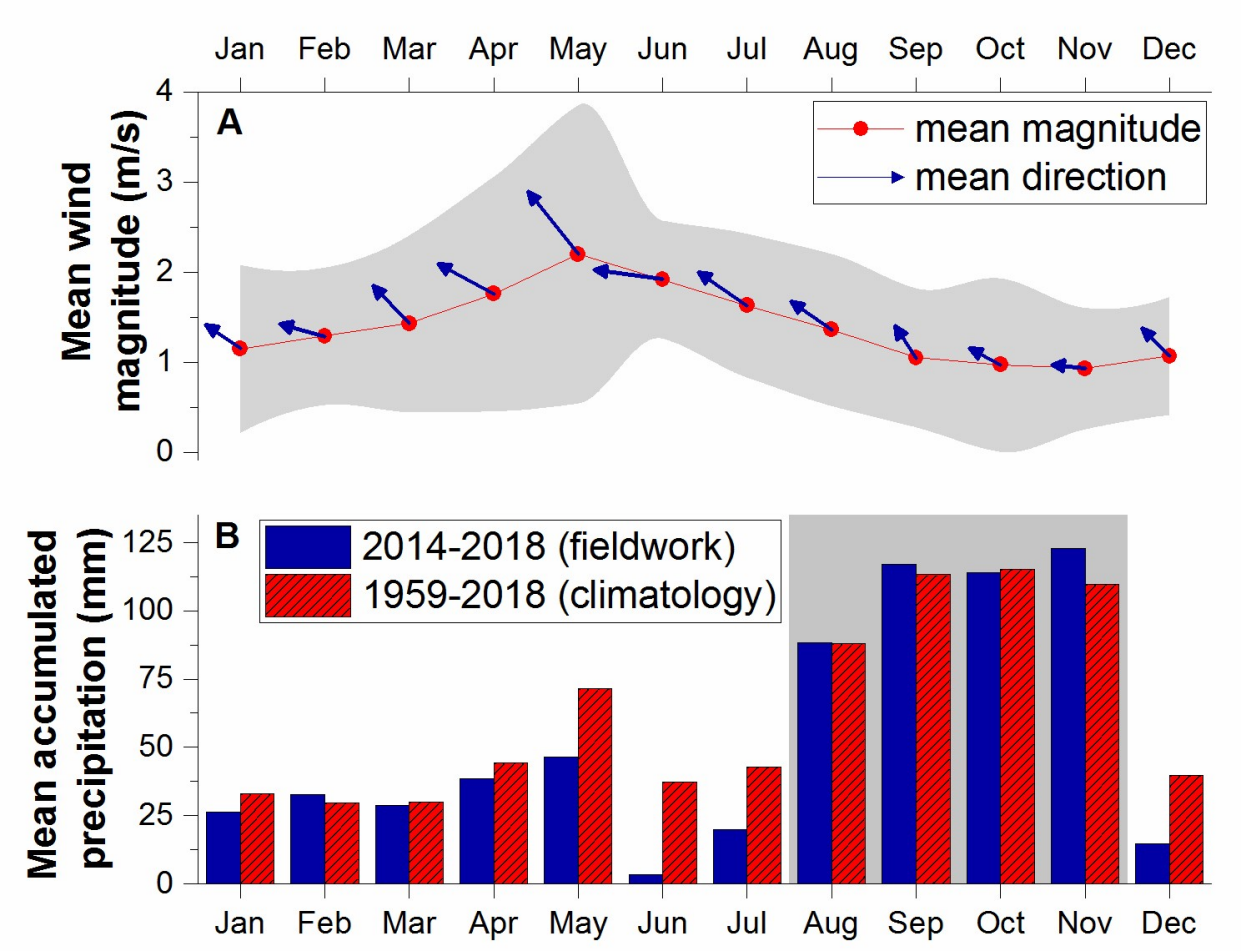
Monthly mean weather conditions at La Parguera. The upper graph (A) shows the mean wind magnitude for each month during the sampling period, where the gray area depicts one standard deviation of the wind magnitude. The lower graph (B) shows the mean accumulated precipitation during the sampling period (blue) and the climatology (red), where the gray area depicts the wet season.

Wind observations (Fig 3A) were obtained from a meteorological station [39] at the UPRM’s Magueyes Island (Fig 1). The station is 3.1 km west of the BB site. We obtained daily accumulated precipitation observations (Fig 3B) collected at Magueyes Island from NOAA’s National Center of Environmental Information [39]. Seasonality at La Parguera is clearly defined by precipitation patterns on a climatological scale and throughout the sampling period (2014–2018). Sea-level data for La Parguera is available from a NOAA tide gauge (station number 9759110) located in the west-end of Magueyes Island [40].

### Carbonate chemistry analyses and calculations

From June 2014 to May 2017, seawater samples for TA were analyzed at the University of New Hampshire (UNH) Ocean Processes Analysis Laboratory. TA analyses followed the Gran titration method [41], using an Apollo SciTech automated titrator and 0.1 N hydrochloric acid (HCl) titrant. The Orion 3-Star pH electrode (Thermo Fisher Inc.) was calibrated using low ionic strength pH buffers (certified on the U.S. National Bureau of Standards Scale to ± 0.01). Dickson’s Certified Reference Materials (CRM) [42] were used to standardize the HCl titrant and validate the results. The precision and accuracy of the TA method as implemented at UNH were ± 2 μmol kg^-1^ and ± 3–4 μmol kg^-1^, respectively. A CRM was analyzed at the beginning and end of each run to correct for any potential drift. From June 2017 to May 2018, TA samples were analyzed at the University of Puerto Rico Mayagüez Campus (UPRM), Department of Marine Sciences, following the method described above and using similar instrumentation. The precision and accuracy of the TA method as implemented at UPRM were ± 2 μmol kg^-1^ and ± 3–4 μmol kg^-1^, respectively. We note that the TA samples from BB may include contributions from organic compounds [43], which we did not account for in this experiment.

All DIC samples were analyzed at UNH by measuring the infrared absorption of the CO_2_ released upon the sample’s acidification with 10% phosphoric acid using an Apollo SciTech AS-C2 automated analyzer. CRM’s were also used to calibrate the instrument. The precision and accuracy of the DIC method as implemented were ± 2 μmol kg^-1^ and ± 5 μmol kg^-1^, respectively.

We calculated the seawater CO_2_ partial pressure (*p*CO_2_), Ω_arg,_ and pH (total scale) from TA and DIC using the CO2SYS v2.1 software [44]. We considered the carbonic acid dissociation constants K_1_ and K_2_ from Lueker [45], the acidity constant of the bisulfate ion from Dickson [46], and the boric acid constant from Uppstrom [47]. For the SE station, we used the boric acid constant from Lee [48], given that it is more suitable for higher pH values. The solubility constant used to derive the Ω_arg_ is from Mucci [49].

### Air-sea CO_2_ flux

The air-sea CO_2_ flux was estimated according to *F = k S ΔpCO*_*2*_, where *F* is the flux in mmol m^-2^ day^-1^, *k* is the gas transfer velocity (m day^-1^), *S* is the CO_2_ solubility coefficient according to Weiss [50], and *ΔpCO*_*2*_ is the ocean surface and atmosphere *p*CO_2_ difference. We obtained the atmospheric *p*CO_2_ values from the Ma*p*CO_2_ buoy in the Enrique Cay forereef (Fig 1). We considered the recent parametrization from Ho [52] that integrates horizontal current velocity, depth, and wind speed to calculate the gas transfer velocity. This parametrization works well for this type of study since it considers any bottom-generated turbulence. We assumed a horizontal current velocity of 5 cm s^-1^ for the latter calculation, given that such magnitude is approximate from previous observations (unpublished results). Wind speed was measured at a 15-meter height and adjusted to a 10-meter wind [51].

### pH drivers and seasonality analysis

To assess the pH seasonality and its controlling mechanisms in BB, we calculated (using CO2SYS) the effects of temperature, salinity, TA, DIC, and air-sea DIC exchange on pH. The following equation describes observed monthly changes between time t_2_ and t_1_ (e.g., February and January) in pH (*ΔpH*), assuming that each process modified the carbonate system independently and that the water column is well mixed:

ΔpH=ΔpH(T)+ΔpH(S)+ΔpH(F)+ΔpH(TA)+ΔpH(DIC)+R (1)

where the terms on the right-hand side of the equation correspond to changes in temperature (T), salinity (S), air-sea DIC exchange (F) in CO_2_ form, TA, DIC, and a residual (R), which represents the remainder of the difference between the terms of the right-hand side of Eq (1) and the observed pH changes (*ΔpH*). Differences in pH were between the initial (*pH*_*calc*_ at t_1_) and modified (*pH*_*2*_ at t_2_) conditions according to the following equations:

$$\Delta pH(T)={pH}_{2}(T_{2},S_{1},{TA}_{1},{DIC}_{1})-{pH}_{calc}$$ (2)

$$\Delta pH(S)={pH}_{2}(T_{1},S_{2},{TA}_{1},{DIC}_{1})-{pH}_{calc}$$ (3)

$$\Delta pH(TA)={pH}_{2}(T_{1},S_{1},{TA}_{2},{DIC}_{1},)-{pH}_{calc}$$ (4)

We refer to *pH*_*1*_*(T*_*1*_,*S*_*1*_,*DIC*_*1*_,*TA*_*1*_*)* at t_1_ as *pH*_*calc*_. Changes in DIC drive both *ΔpH(F)* and *ΔpH(DIC)*: the former term refers to DIC changes resulting from air-sea CO_2_ exchange, while the latter term includes DIC changes caused by both water-column and benthic mechanisms. To determine *ΔpH(F)*, we first calculated the DIC change (*ΔDIC*_*air-sea*_) using the CO_2_ air-sea exchange (*F*, mmol m^-2^ d^-1^), seawater density (*d*, kg m^-3^), change in time (*t*_*2*_*-t*_*1*_, days), and sampled depth (*H*, m) [52]:

$$\Delta {DIC}_{air-sea}=\frac{-F\cdot (t_{2}-t_{1})}{d\cdot H}$$ (5)

$$\Delta pH(F)={pH}_{2}(T_{1},S_{1},{TA}_{1},{{DIC}_{1}+\Delta DIC}_{air-sea})-{pH}_{calc}$$ (6)

Given that changes in pH due to DIC include the effect of CO_2_ air-sea exchange, we subtracted *ΔpH(F)* from *ΔpH(DIC)* to separate both signals:

$$\Delta pH(DIC)=({pH}_{2}(T_{1},S_{1},{TA}_{1},{DIC}_{2})-{pH}_{calc})-\Delta pH(F)$$ (7)

## Results and discussion

### Carbonate chemistry spatio-temporal variability

**Fig 4:**
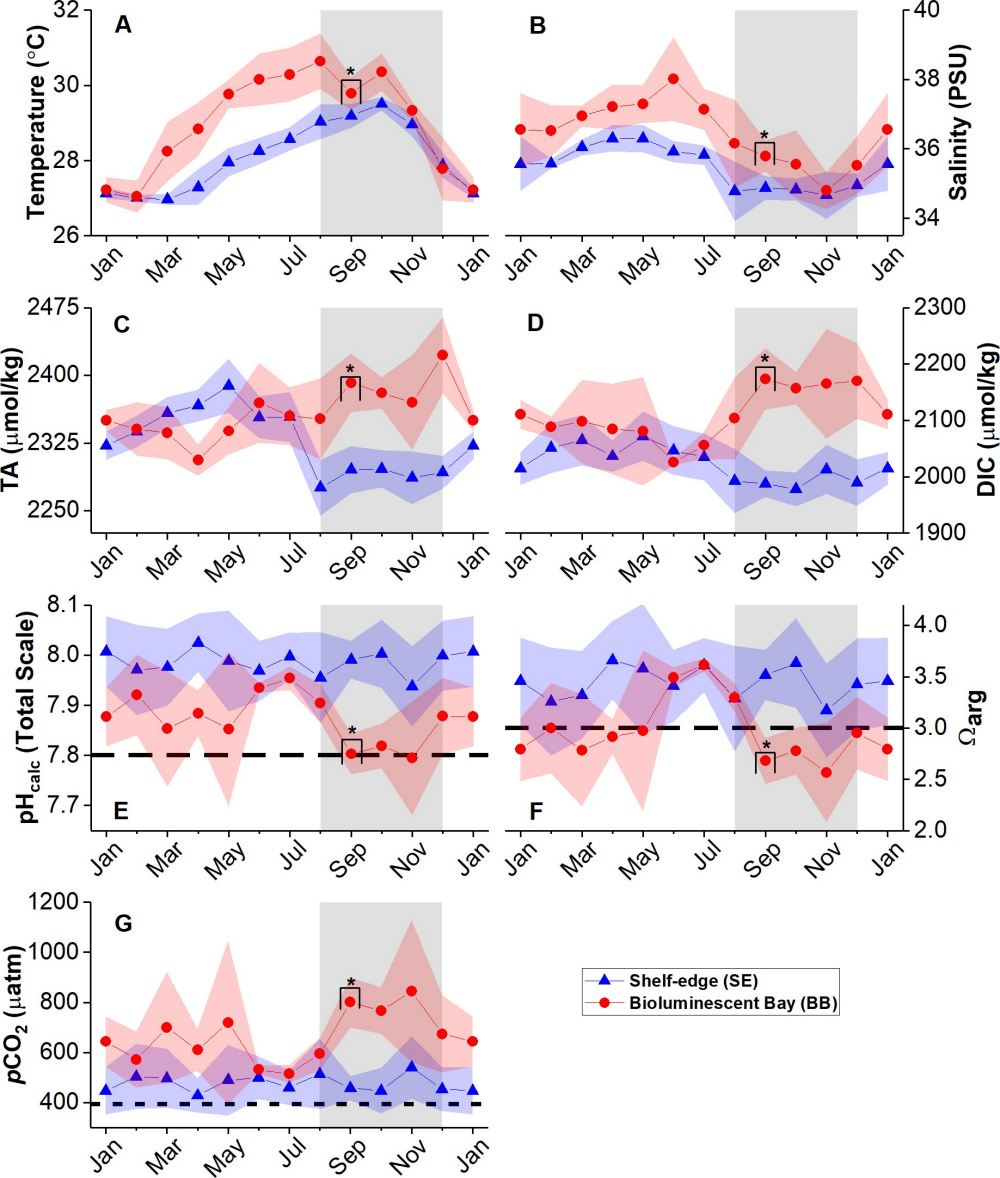
Monthly mean carbonate chemistry parameters at BB and SE stations. (A) Monthly mean temperature, (B) salinity, (C) TA, (D) DIC, (E) pH_calc_, (F) Ω_arg_ and (G) *p*CO_2_. The long-dashed lines in (E) and (F) represent expected mean values by 2100 under business-as-usual scenarios (RCP 8.5) [55]. The short-dashed line in (G) is the mean atmospheric *p*CO_2_ value (394 μatm) during the sampling period. Red and blue shaded areas represent one standard deviation for each data point, while gray shades represent the wet season. For every averaged data point, n ≥ 3. Data points depicted with an asterisk show where n = 3. Data are in S1 and S2 Tables.

We averaged all the interannual data from BB and SE to monthly values (Fig 4). Differences in salinity and temperature variabilities between BB and SE support the contention of limited exchange between BB and outer coastal waters [27, 53]. The exchange regime results from the semi-enclosed physiography and the small tidal range typical for the region. The larger magnitudes of temperature and salinity observed at BB are likely due to higher daytime evaporation rates [54]. Water temperature remained above 29 ^o^C at BB for most of the year, from May to November. We detected the salinity minimums at both stations in November due to large amounts of accumulated precipitation (Fig 3B). The salinity decrease at SE after April was due to the influence of riverine water from the Amazon and Orinoco river plumes, later magnified by the maximum outflow of the Orinoco in September [29, 30] and the start of the wet season in August (Fig 3B). June was the driest month during the sampling period and anomalously dry relative to climatological data. BB’s higher salinities are due to its enclosed nature and the year-round dominance of evaporation [27] and evapotranspiration [54] over precipitation.

The data presented in Table 1 and Fig 4 show that BB was consistently more acidified (lower pH and Ω_arg_) than SE throughout the year. The larger amplitudes observed in the BB data evidence a marked seasonality relative to the SE data. BB’s pH values are near the projected 7.8 mean for 2100 under business-as-usual CO_2_ scenarios (RCP 8.5) during September and November, while we often observed Ω_arg_ below or near the 3.0 projected value [55].

**Table 1:** Maximum and minimum monthly means (± one standard deviation) for each parameter from Fig 4.

Observations	SE			BB		
	*Mean*	*Maximum*	*Minimum*	*Mean*	*Maximum*	*Minimum*
**Temperature**	28.19	29.51	26.97	29.03	30.64	27.04
**(**^**o**^**C)**	± 0.93	± 0.18	± 0.14	± 1.36	± 0.74	± 0.42
**Salinity**	35.42	36.31	34.66	36.47	38.01	34.81
	± 0.74	± 0.38	± 0.68	± 1.14	± 1.21	± 0.54
**TA**	2323	2389	2275	2359	2407	2306
**(μmol kg**^**-**^**^1^)**	± 42	± 30	± 31	± 43	± 57	± 17
**DIC**	2020	2072	1981	2114	2175	2035
**(μmol kg**^**-**^**^1^)**	± 49	± 44	± 29	±73	± 108	± 38
**pH**_**calc**_	7.99	8.02	7.94	7.87	7.96	7.79
**(total scale)**	± 0.07	± 0.05	± 0.08	± 0.09	± 0.02	± 0.11
**Ω**_**arg**_	3.45	3.67	3.17	2.96	3.64	2.57
	± 0.43	± 0.35	± 0.46	± 0.47	± 0.07	± 0.48
***p*CO**_**2**_	477	540	430	674	845	497
**(μatm)**	± 101	± 125	± 63	± 188	± 284	± 39

BB’s lower pH values coincided with higher TA concentrations (relative to SE), suggesting that the excess TA was insufficient to buffer the declines in pH and Ω_arg_. Thus, we observed an average excess (relative to the oceanic reference station, SE) of mean TA and DIC of + 36 and + 94 μmol kg^-1^, respectively. The mean seawater *p*CO_2_ (Fig 4G) at BB station was above the mean atmospheric *p*CO_2_ (400 μatm) year-round by approximately 270 μatm.

As indicated by pH and Ω_arg_ data, the acidification reaches its maximum at BB during the wet season when the mean pH decreased by 0.06 units, and the mean Ω_arg_ decreased by 0.19. During that time (Aug-Nov), we observed an excess of mean TA and DIC of + 81 and + 152 μmol kg^-1^, respectively, relative to SE. Thus, mean TA and DIC in BB increased during the wet season by 15 and 48 μmol kg^-1^, which is not enough to induce a net buffering effect [7]. Also, we observed *p*CO_2_ values above 600 μatm, thus 200 μatm above the 400 μatm atmospheric global means. This study provides the first characterization of the seasonal carbonate chemistry within a mangrove system in Puerto Rico. These results show that not all mangrove lagoons can buffer OA at seasonal time scales and serve to advance our understanding of the diverse roles mangroves may play in modulating acidification in tropical coastal environments.

### Influence of TA and DIC variability on pH and Ω_arg_

**Fig 5:**
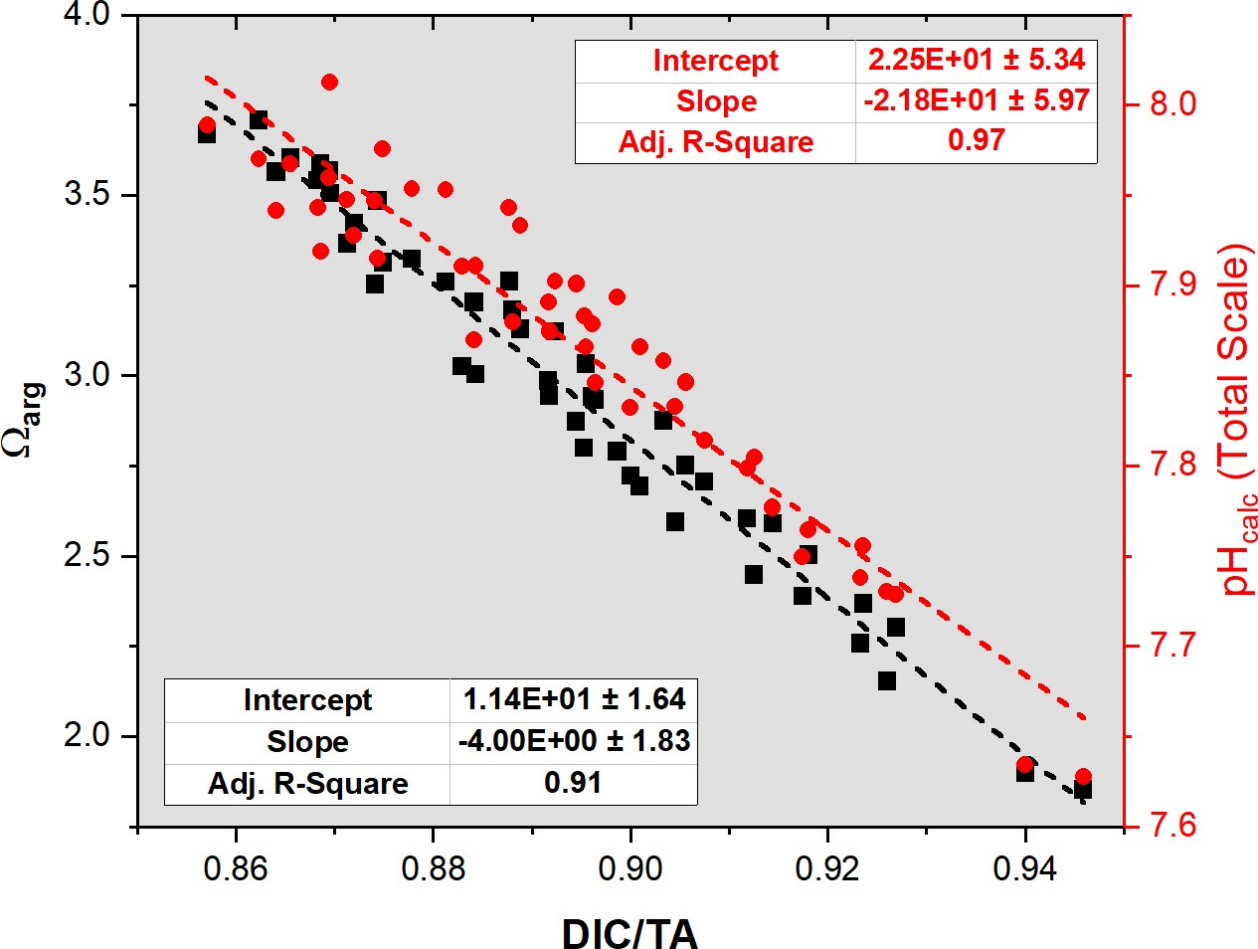
Linear correlation of the DIC/TA ratio with pH and Ω_arg_.

The relationship between DIC and TA can provide insights into the mechanisms that control OA [56]. Both Ω_arg_ and pH_calc_ showed significant (*p* < 0.00001; n = 47) and strong correlations (R^2^ = 0.97 and 0.91, respectively) with the DIC/TA ratio (Fig 5). This ratio evidences the sensitivity of the system to perturbations in dissolved seawater CO_2_ concentration [57]. If the DIC to TA ratio equals 1, any addition/removal of CO_2_ (in the form of DIC) will result in a maximum decrease/increase of seawater pH and Ω_arg_ [58], with soluble CO_2_ being more sensitive to temperature changes. Thus, TA and DIC changes controlled the pH decrease and Ω_arg_ we observed in BB during the wet season (Fig 4).

When transitioning from the peak of the dry season (June-July) to the wet season (August-November), the mean DIC/TA ratio increased significantly (*p* < 0.001, n = 6 and 16, respectively) from 0.87 ± 0.00 to 0.91 ± 0.02. At this time, we observed increases in DIC and TA of 100 and 6 μmol kg^-1^, respectively, decreasing pH and Ω_arg_ by 0.12 and 0.75. Thus, the observed changes in water chemistry evidence that the increase of CO_2_ production (presumably from benthic and water column aerobic respiration) surpassed the increase of TA production from anaerobic processes, suggesting aerobic dominance over anaerobic remineralization. Sulfate reduction is an important TA producing reaction in tropical mangrove sediments [7, 21, 59, 60], while denitrification is not [7, 24, 61, 62]. Previously measured water column nitrate concentrations at BB were low (0.12 μmol L^-1^, annual mean) and showed a limited seasonal variability [35]. Another study [24] in La Parguera reported low benthic denitrification (0.03–0.35 μmol m^-2^ h^-1^) and nitrification (0.44–3.15 μmol m^-2^ h^-1^) rates. However, we cannot rule out denitrification from occurring in the water column or the sediment–seawater interface. During the wet season, the salt flats to the north get flooded, and water flows into BB, possibly exporting nitrate and further enhancing denitrification [63].

Previous research (S3 Table) at BB reported that water column respiration exceeds primary production year-round. Aerobic respiration of organic matter releases essentially unbuffered DIC [9], given that it increases DIC by 1 mol and decreases TA by 0.2 moles for each mol of carbon oxidized (assuming a Redfield ratio). Previous work in mangrove environments has documented an increase in heterotrophic activity during the wet season [58, 61, 64], possibly fueled by carbon derived from allochthonous sources (i.e., phytoplankton, seagrass, terrestrial non-mangrove, or direct input of TA and DIC from runoff) or local sources (i.e., water column mechanisms or benthic mechanisms) [22, 59].

Studies have observed elevated concentrations of sulfide at bottom waters (1.6–3.5 mmol L^-1^) [32] and in porewaters (0.024–3.2 mmol L^-1^) [37] throughout the year. High sulfate reduction rates in BB (34.06 mmol L^-1^ h^-1^) relative to an oceanic station located ca. 10 km from shore (9.33 mmol L^-1^ h^-1^) were reported as well [37]. The higher sulfate reduction rates observed (see [37]) in BB indicate its potential role as a TA source, assuming minimal re-oxidation of reduced sulfur species [65, 66]. Sulfate reduction produces one mole of TA per mole of organic carbon oxidized, assuming that the sulfur species are not re-oxidized to sulfate [67].

### The drivers of the pH seasonality

**Fig 6:**
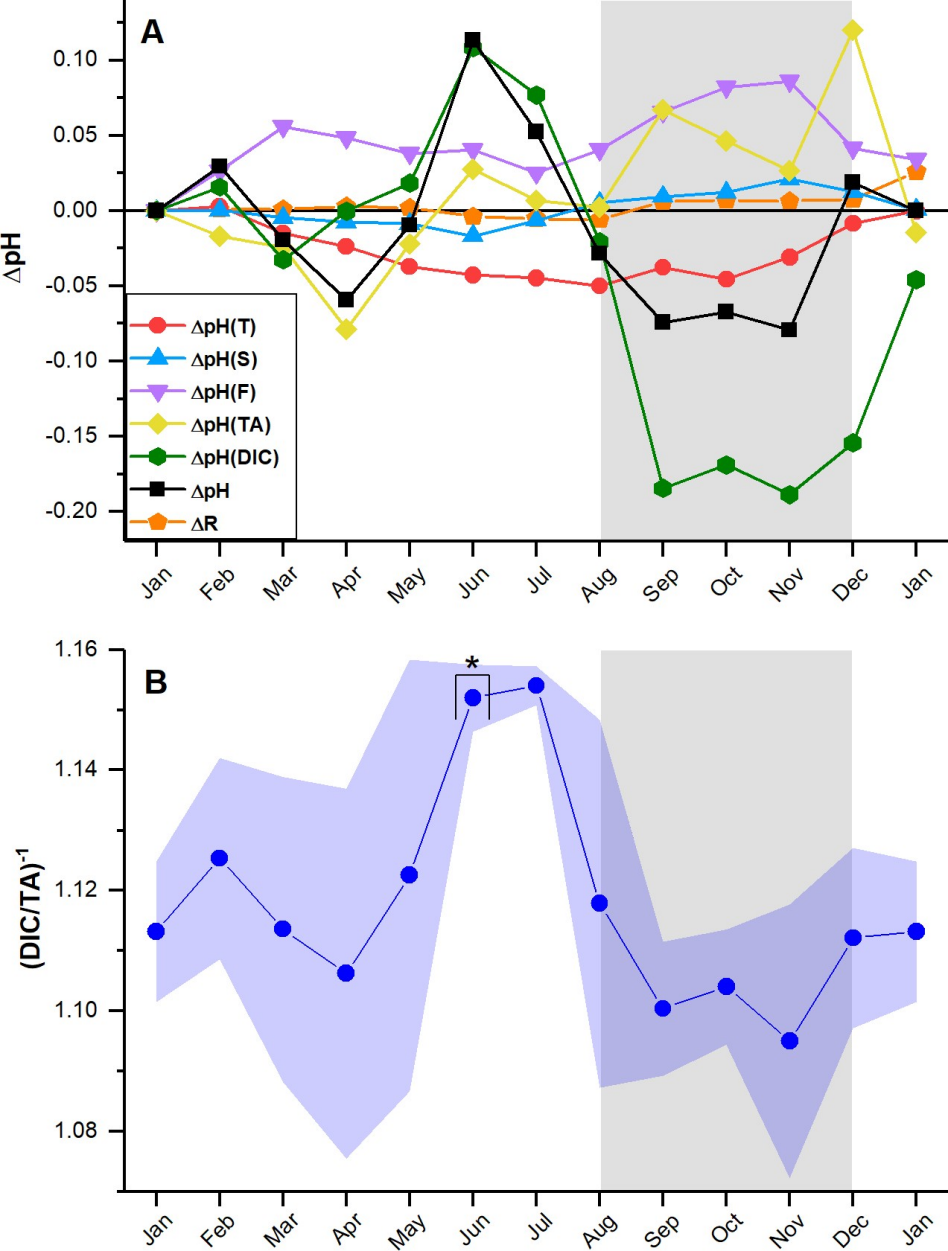
Results of the pH decomposition. (A) Cumulative monthly changes in pH (black line) due to temperature (red), salinity (blue), CO_2_ flux (violet), TA (yellow), and DIC (green). The analysis considered January as the starting point and added the changes from thereon. (B) Seasonal variability of the inverse DIC/TA ratio. The blue shade represents one standard deviation for each monthly mean. For each data point, n ≤ 2. Data points depicted with an asterisk show where n = 2. The gray shades in both plots represent the wet season.

The pH decomposition (Fig 6A) shows that seasonal changes were influenced the most by biological processes, given that *ΔpH(DIC)* and *ΔpH(TA)* exerted the most considerable changes to monthly pH. The DIC/TA ratio (Fig 6B) closely follows the observed pH changes (ΔpH). Thus, the balance between changes in DIC and TA mainly modulated the pH variability. Increases in DIC decrease pH, while increases in TA increase pH. The mean annual magnitudes of *ΔpH(T)* and *ΔpH(S)* together equal 0.04, while the mean magnitudes of *ΔpH(DIC)* and *ΔpH(TA)* equal 0.08 and 0.04, respectively. Therefore, changes in pH due to mixing, dilution, and evaporation were the least significant. With a mean annual magnitude of 0.05, air-sea CO_2_ exchange increased pH (*ΔpH(F)*) year-round as expected [21]. When comparing the TA at BB with the TA at SE from March to May, we observe that the TA depleted in BB. Thus, some local processes must have consumed alkalinity. During the wet season, we observed the largest decrease in *ΔpH(DIC)* and increase in *ΔpH(TA)*, confirming that CO_2_ production from (presumably) aerobic respiration and TA production from anaerobic respiration increased at this time, while aerobic respiration dominated.

## Summary and conclusions

Results from this study evidence that BB could not buffer OA over a seasonal time frame. Although TA increased throughout the year, it was not enough to buffer the observed declines in pH and Ω_arg_ resulting from increases in DIC. This acidification effect exacerbated during the wet season due to increased precipitation coinciding with more extensive tidal excursions (range), presumably enhancing the transport of labile organic matter into BB. Such an increase in heterotrophic activity during the wet season seems to be a general feature at La Parguera [68] enhanced in BB.

Given the insight gained in this study, future research should integrate temporal and diel surveys to better assess carbonate chemistry variability and trends at various time scales. It is unknown if the observed acidic conditions export to nearby environments (and to what extent) or if such conditions are detrimental to organisms other than corals. Future studies should couple the rates of benthic and water column remineralization processes with in-situ carbonate chemistry measurements. The chemistry and nutrient concentrations of runoff water should be measured as well. It is also essential to assess how the acidification discussed can impact organisms that live in the mangrove root systems and sediments.

## Supporting information

S1 Fig Mean tide level seasonality in meters at La Parguera. Data frequency is one hour and corresponds to the sampling period (2014–18, top to bottom). Data are shown in blue, while the red line represents a third-degree polynomial fitting for each dataset. Gaps during the latter part of 2017 and the beginning of 2018 are due to the mareograph malfunctioning after Hurricane María (September 2017).
(TIF)

S1 Table Mean monthly data for all parameters measured at BB. (XLSX)

S2 Table Mean monthly data for all parameters measured at SE. (XLSX)

S3 Table Water column metabolic rates previously measured at BB. (PDF)

S1 Dataset Data corresponding to the BB station. (CSV)

S2 Dataset Data corresponding to the SE station. (CSV)
