# Supplementary figures and images for: Carbonate chemistry seasonality in a tropical mangrove lagoon in La Parguera, Puerto Rico
Source: PLoS One. 2021 May 5;16(5):e0250069. doi: 10.1371/journal.pone.0250069 (PMC8099052; doi:10.1371/journal.pone.0250069)

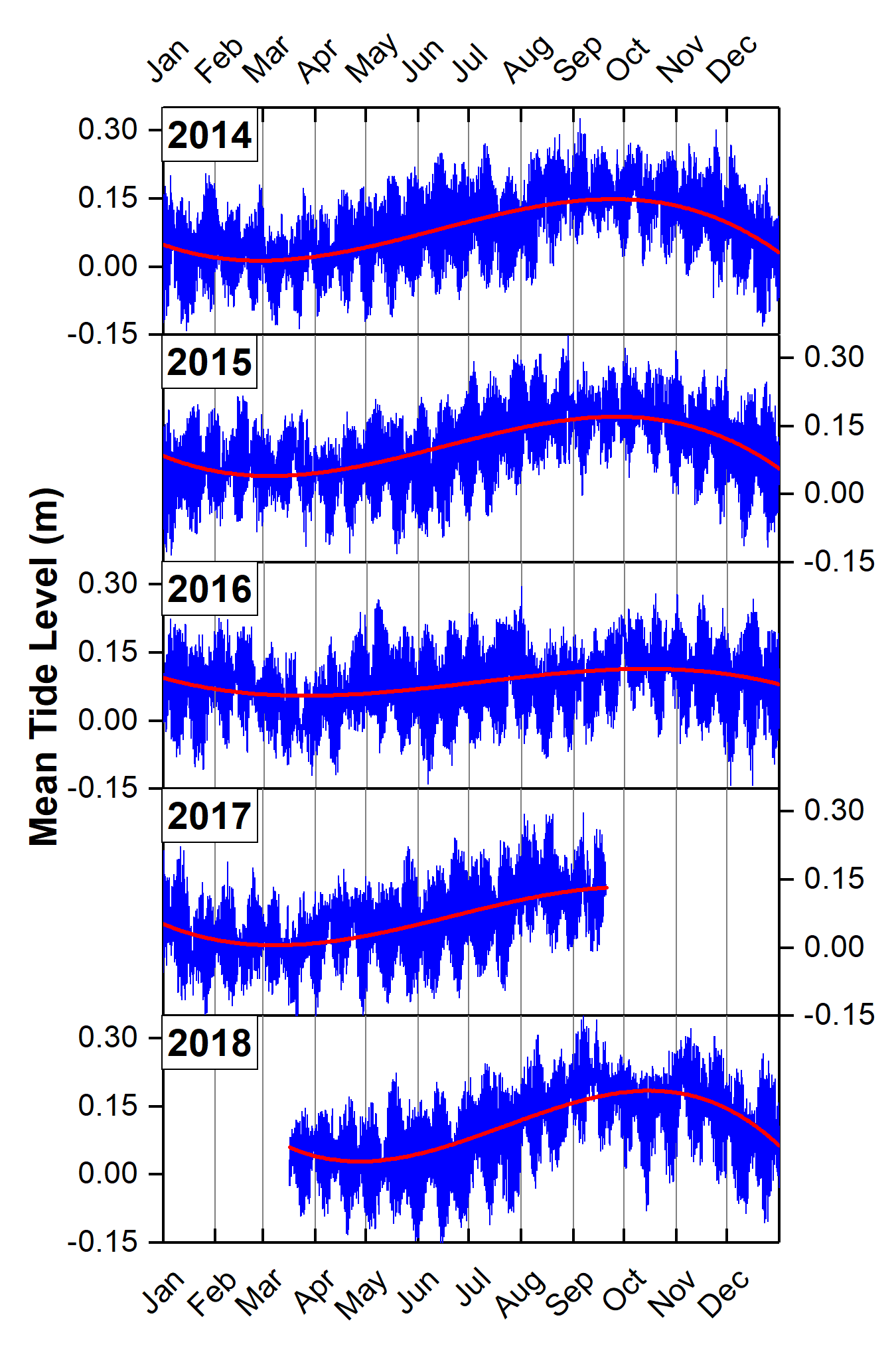

Supplement: S1 Fig — Data frequency is one hour and corresponds to the sampling period (2014–18, top to bottom). Data are shown in blue, while the red line represents a third-degree polynomial fitting for each dataset. Gaps during the latter part of 2017 and the beginning of 2018 are due to the mareograph malfunctioning after Hurricane María (September 2017). (TIF) [file pone.0250069.s001.tif]
