# Supplementary material for: Carbonate chemistry seasonality in a tropical mangrove lagoon in La Parguera, Puerto Rico
Source: PLoS One. 2021 May 5;16(5):e0250069. doi: 10.1371/journal.pone.0250069 (PMC8099052; doi:10.1371/journal.pone.0250069)
Supplement: S3 Table — (PDF) [file pone.0250069.s004.pdf]

Water column metabolism						
Reference	*Production*	*Respiration*	*n*	*Scale*	*Method*	*Notes*
[1]	0.00 - 35.20	2.49 - 41.19	7	Seasonal	Winkler	g m^{-2} day^{-1}
[1]	0.93 - 5.89	9.61 - 17.05	2	Diurnal	Winkler	g m^{-2} day^{-1}
[2]	0.06 - 0.79	(no data)	10	Seasonal	C-14	g m^{-3} day^{-1}
[3]	1.40	2.32	1	Diurnal	Winkler	g m^{-2} day^{-1}

References:

1. Cintrón-Molero G. Seasonal fluctuations in a tropical bay [master's thesis]. [Mayagüez (PR)]: University of Puerto Rico at Mayagüez; 1969. 111 p.

2. Gonzalez JG. Primary productivity of the neritic and offshore waters of western Puerto Rico. Mayagüez (PR): Institute of Marine Sciences; 1965. 40p. Contract No.: NONR-4318(00). Supported by U.S. Office of Naval Research. 1967.

3. Odum HT, Burkholder PR, Rivero J. Measurements of productivity of turtle grass flats, reefs and the Bahia Fosforescente of southern Puerto Rico. Publ Inst Mar ScI Univ Texas. 1959; 6: 159-170.
